# Therapeutic Advancements in the Management of Psoriasis: A Clinical Overview and Update

**DOI:** 10.7759/cureus.79097

**Published:** 2025-02-16

**Authors:** Sarah F Qasem, Hasan Ashkanani, Ali Ali

**Affiliations:** 1 Dermatology, Amiri Hospital, Ministry of Health, Kuwait City, KWT; 2 Internal Medicine, Mubarak Al-Kabeer Hospital, Jabriya, KWT

**Keywords:** biologics, management, nails, psoriasis, skin

## Abstract

Psoriasis is an autoimmune chronic inflammatory skin condition with a strong genetic predisposition. Pathogenesis of psoriasis is complex and multifactorial; it is known that genetic, immunological, and environmental factors play significant roles in its development. Treatment options vary and include topical therapy (e.g., corticosteroids, vitamin D analogs, and calcineurin inhibitors), phototherapy (e.g., narrowband ultraviolet radiation (NB-UVB)), and systemic therapy (e.g., methotrexate and retinoids).

Several new treatments have emerged in recent years, including biological treatments. Biologics approved by the United States Food and Drug Administration (FDA) for the treatment of psoriasis include inhibitors of tumor necrosis factor (TNF)-α. Other FDA-approved biologics for the treatment of psoriasis target cytokines, such as the p40 subunit of interleukin (IL)-12 and IL-23, IL-17, as well as the p19 subunit of IL-23. Additionally, the Janus kinase (JAK) inhibitor deucravacitinib is also FDA-approved for the treatment of moderate-to-severe psoriasis. Other promising treatment modalities are consistently undergoing trials.

Further therapeutic details, including regimens, side effects, indications, contraindications, and FDA approval dates, are discussed comprehensively in this article. For the purpose of this review, the literature was thoroughly searched for publications discussing psoriasis therapy. This review aims to provide a comprehensive overview and update on the management of psoriasis.

## Introduction and background

Psoriasis is an autoimmune inflammatory skin condition [1], affecting millions of people globally and potentially causing psychological and financial burdens. There are several types of psoriasis known, including chronic plaque psoriasis, pustular psoriasis, erythrodermic psoriasis, and guttate psoriasis. Psoriasis can also affect the nails and scalp, and in some cases, the joints, leading to psoriatic arthritis. Symptoms vary among individuals, and the treatment of choice depends on the severity of the disease or the type of psoriasis. In the past couple of years, several treatments have emerged, while many other promising treatment modalities are undergoing trials [2]. There have been significant advancements in the treatment of psoriasis. This review aims to provide a comprehensive overview and update on the management of psoriasis. 

The literature was thoroughly searched for English-language publications containing the keywords "psoriasis treatment" or "biological psoriasis treatment". Articles discussing psoriasis therapy were selected for further review, with an emphasis on recent studies and those detailing novel therapies. Randomized, double-blind, placebo-controlled trials and meta-analyses were particularly sought, although other types of studies, including open-label trials and case series, were also reviewed. Exclusion criteria included articles more than 20 years old and journals with consistently low scientific impact. Selection and publication bias were minimized through serial author review, though some minimal bias may remain.

## Review

Psoriasis is a chronic immune-mediated inflammatory skin condition with a strong genetic predisposition and an autoimmune pathogenic trait. Its worldwide prevalence is up to 2% but may vary according to region [1]; the prevalence of psoriasis is known to be higher in colder climates compared to tropical climatic regions. The human leukocyte antigen HLA-Cw6 is the most commonly associated genetic factor in psoriasis, especially in early-onset psoriasis. Located on chromosome 6p21, it plays a critical role in immune regulation and increases susceptibility to autoimmune responses. Another gene, CARD14, is particularly linked to familial cases of psoriasis and severe subtypes of psoriasis, including generalized pustular psoriasis.

Clinical manifestations vary as per the course, severity, and triggers. Although psoriasis can occur at any age, studies suggest that it has a bimodal distribution with respect to the age of onset; the first peak affects younger adults between 20-30 years of age and the second peak affects the middle-aged population between 50-60 years [2]. This condition affects the skin mainly, but nails and joints are also commonly involved. Psoriasis primarily affects the extensor surfaces of the skin, although other areas may also be involved, including the palms and soles, the scalp, as well as the genital area [3]. Skin lesions are usually symmetrical, sharply demarcated, with red or pink raised plaques, and covered with silvery scales that can flake off and are itchy; sometimes, lesions may even cause pain. Further, in other cases, if an adherent scale is removed, pinpoint bleeding may be evident, also known as the Auspitz sign. Psoriasis is diagnosed based on physical examination and thorough history taking; laboratory and/or histopathologic confirmations are rarely needed, though exceptions that require histopathologic confirmations do exist and include atypical presentation, isolated nail psoriasis, and resistance to treatment. As previously mentioned, involvement of the nails is common and affects 80-90% of patients with plaque psoriasis, usually presenting as nail pitting, onycholysis, and sometimes, subungual hyperkeratosis. Nail involvement is even more common in patients with psoriatic arthritis.

The pathogenesis of psoriasis is complex, involving genetic, environmental, and immunological factors. Inflammation in psoriasis is primarily driven by T-helper (Th) 17 and Th1 cells and is mediated by cytokines such as tumor necrosis factor (TNF)-⍺, interleukin (IL)-17, and IL-23. In addition, there is abnormal keratinocyte hyperproliferation, where cytokines released from activated T-cells trigger the overproduction of keratinocytes, leading to thickened skin, as seen in psoriasis. A loss of normal differentiation is also observed [4]. Angiogenesis plays a role in psoriasis as well, increasing the blood supply to affected areas with greater vascular permeability, which allows more cytokines to enter the skin, further driving inflammation and vascular endothelial cell proliferation.

Psoriasis is associated with a number of comorbidities, including psoriatic arthritis, cardiovascular diseases, and other non-communicable diseases (NCDs), such as diabetes and hypertension [5]. Also, psoriasis patients are commonly reported to have psychiatric diseases, such as depression and generalized anxiety disorders, and may have an increased risk of suicidal ideation [5].

Psoriasis is an incurable condition, and treatment aims to manage symptoms and improve the quality of life. The first step in the treatment of psoriasis is identifying and addressing common comorbidities that may already exist or may develop in the future, such as joint involvement. Treatment focuses on controlling symptoms and is often life-long. Therefore, it should not only be efficacious but also safe for longer periods and affordable. Recognizing triggers is a major part of psoriasis management. Stress, tobacco use, infections, alcohol use, hormones, skin trauma, and medications have been implicated in triggering the first occurrence or subsequent flare-ups of psoriasis [6]. Medications that may trigger the first episode or subsequent flare-ups of psoriasis include lithium, B-blockers (BB), calcium channel blockers (CCB), angiotensin-converting enzyme inhibitors (ACEI), anti-malarials, non-steroidal anti-inflammatory drugs (NSAIDs), and checkpoint inhibitors.

Lifestyle changes such as smoking cessation, weight loss, and minimizing alcohol use have been found to be effective, with a positive impact on the severity of psoriasis. There are three major forms of psoriasis treatment, namely topical therapy, phototherapy, and systemic therapy. Several methods are used to determine the severity of psoriasis, such as calculating the affected total body surface area (BSA) [7]. A BSA score of 3% is considered mild, whereas a score of 3-10% is considered moderate, and above 10% is considered severe. The Psoriasis Area and Severity Index (PASI) score is more specific in evaluating the extent and severity of psoriasis because it takes into account redness, scaling, and plaque thickness, in addition to BSA. It gives a numerical score between 0 and 72, representing no disease to maximal disease, respectively [8]. Physician's Global Assessment (PGA) is an overall estimate of psoriasis severity, ranging from clear to very severe (clear, nearly clear, mild, moderate, severe, and very severe).

Treatment is primarily based on the severity of the disease. For patients with mild psoriasis, topical agents remain the mainstay of treatment. Phototherapy is used when the results of topical agents are not satisfactory. In moderate-to-severe psoriasis, biological therapy is recommended, which includes a group of medications that target specific key receptors involved in psoriasis pathogenesis. Sometimes, in addition to systemic therapy, adding topical treatments directly to lesions may improve the symptoms and outcomes. The American Academy of Dermatology National Psoriasis Foundation guidelines recommend biologics as an option for first-line treatment in moderate-to-severe psoriasis because of their efficacy in treating this condition and acceptable safety profiles [9]. Over the past year, several successful therapies have been approved by the FDA for the treatment of psoriasis, which are discussed in detail in the next sections.

General measures

Patients with psoriasis should be advised to use an emollient on a daily basis. This improves the skin barrier, relieves itching, and reduces fissuring. Additionally, emollients may increase the efficacy of topical corticosteroids by improving their penetration through the skin layers [10]. Furthermore, current guidelines recommend keratolytics, such as topical preparations containing urea and salicylic acid, as adjuvant therapy for psoriasis across all severities [11]; this method significantly reduces the amount of scale, thereby enhancing the penetration of active ingredients into the skin. 

Topical therapy (the first-line treatment for localized mild psoriasis)

Topical Corticosteroids

**Figure 1:**
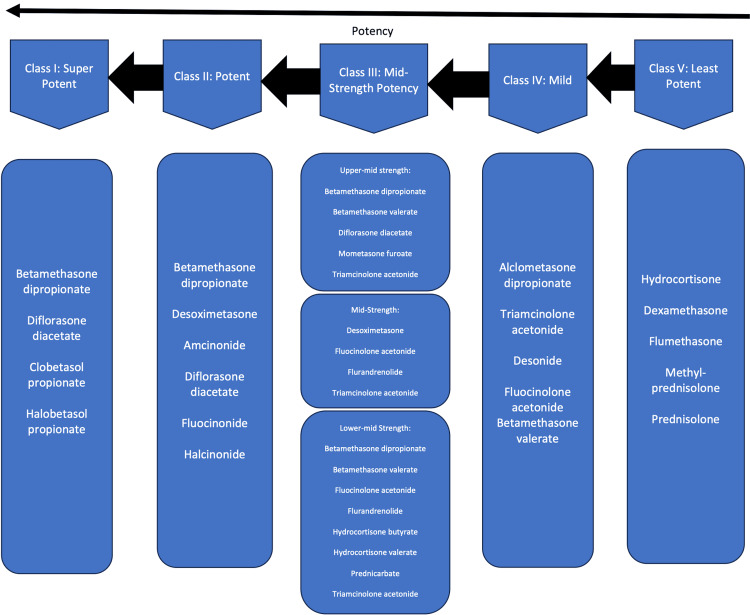
Topical corticosteroid potency chart Topical corticosteroid classification based on potency, with class 1 being the most potent. Credits: Author Hasan Ashkanani

Topical corticosteroids vary in potency, from low to medium to high potency (Figure 1). Low-potency formulations are used on the face and genital areas, whereas higher-potency corticosteroids are used for thicker plaques, such as those found on the body or the scalp. In some cases, rapid relapse, such as in the pustular form of psoriasis, occurs. If necessary, short-term use of a more potent corticosteroid on sensitive areas is acceptable; however, the clinician should weigh the benefits and risks for each patient individually. 

Patients are usually very compliant with topical corticosteroids due to their cosmetic effect, but potential side effects are probable without adequate supervision. Refractory psoriasis, especially on the hands and/or feet, requires high-potency corticosteroids, sometimes under occlusion. Side effects of long-term use of corticosteroids include corticosteroid-induced cutaneous atrophy and telangiectasia, particularly on the face. Systemic side effects are rare and include Cushing’s syndrome. Tachyphylaxis is a common side effect of topical corticosteroids and refers to an acquired diminished response to a drug following repeated use over a short period. This phenomenon is most frequently associated with high-potency topical steroids. To prevent tachyphylaxis, corticosteroids should be used intermittently or for limited durations, often in combination with steroid-sparing agents. 

Topical Vitamin D Analogs

Vitamin D analogs currently available include calcipotriol, calcitriol (the active metabolite of vitamin D), and tacalcitol. They are used for the treatment of psoriasis in adults and children aged two years and older. Topical vitamin D3 analogs help clear psoriasis within a few weeks and are cosmetically acceptable, effective, and safe. However, irritation of perilesional skin has been reported as a side effect. Vitamin D analogs improve psoriasis but work slowly. Therefore, combining a topical corticosteroid with a vitamin D analog speeds up the rate of disease improvement, and the patient can see results within one week. This method also reduces irritation and minimizes the need for a higher-potency topical corticosteroid. For maintenance therapy, it has been found that a topical vitamin D analog used daily with a topical corticosteroid applied on the weekends, for instance, is a good management plan to avoid the risk of long-term use of corticosteroids. There is an available combination cream of betamethasone and calcipotriene, which may help patients adhere to the management plan.

Calcineurin Inhibitors

Calcineurin inhibitors, also known as tacrolimus/pimecrolimus, are steroid-free options used for long-term treatment. Unlike topical corticosteroids, they do not have undesired cutaneous adverse effects such as skin atrophy. The most common side effect is a burning or tingling sensation at the site of application. Their use is limited to intertriginous areas. Pimecrolimus is FDA-approved for adults and children over the age of two, whereas tacrolimus has two formulations. Tacrolimus ointment 0.03% is for use in children aged 2-15 years, whereas for adults aged 16 and older, either 0.03% or 0.1% can be used.

Topical Tapinarof 

Tapinoraf 1% cream is a newly FDA-approved treatment for plaque psoriasis. It is a topical aryl-hydrocarbon receptor (AhR) modulating agent derived from a secondary metabolite produced by a bacterial symbiont of entomopathogenic nematodes [12]. AhRs are involved in gene expression regulation, and their activation leads to the inhibition of pro-inflammatory pathways implicated in psoriasis (Th17 pathway; cytokines: IL-17A, IL-17F) [13]. In addition, tapinarof suppresses keratinocyte hyperproliferation and modulates skin-barrier proteins, namely filaggrin and loricrin. Tapinoraf cream is used in adults with plaque psoriasis and atopic dermatitis, regardless of disease activity, and it is the first FDA-approved steroid-free cream. 

The efficacy and safety of tapinarof 1% cream applied once daily has been demonstrated in a comprehensive phase 3, 12-week psoriasis pivotal trials program (PSOARING 1, PSOARING 2, and PSOARING 3) [14-16]. The goal of these trials was to assess long-term safety, efficacy, remittive effects, durability of response, and tolerability of tapinarof cream used once daily. It was found to be significantly more efficacious than vehicle cream and was well tolerated. Efficacy improved beyond the 12-week trial, with a 40.9% complete disease clearance rate [14]. The most common adverse effects were folliculitis, contact dermatitis, pruritus, and headaches [15].

The two phase 3 randomized trials of tapinarof were conducted in patients with mild-to-severe plaque psoriasis, with 510 and 515 patients being enrolled in PSOARING trial 1 and trial 2, respectively. Patients with a baseline PGA score of 2 (mild) to 4 (severe) on a scale of 0-4, and a PASI of 3-20%, were randomly assigned in a 2:1 ratio to use either tapinarof cream or the vehicle cream once daily for 12 weeks. The primary endpoint was defined as a PGA score of 0-1 (clear or almost clear) and a decrease in the baseline score by at least two points at week 12. In the first trial, a PGA response occurred in 35.4% of patients using tapinarof and in 6% of those using the vehicle treatment. In the second trial, the PGA response was 40.2% and 6.3%, respectively. Both comparisons were statistically significant (P<0.001). Adverse events reported with tapinarof included folliculitis, contact dermatitis, headache, upper respiratory tract infections, and pruritus. In conclusion, tapinarof 1% cream, used once daily, was found superior to vehicle cream in reducing the severity of plaque psoriasis over a period of 12 weeks. 

Topical Roflumilast

Another newly FDA-approved cream for the treatment of plaque psoriasis is roflumilast 0.3%, a selective, topical, highly potent phosphodiesterase (PDE)-4 inhibitor, that was also found to be efficacious and safe in the treatment of plaque psoriasis. Inhibition of PDE-4 decreases the expression of pro-inflammatory cytokines implicated in the pathogenesis of plaque psoriasis, such as TNF-⍺, interferon (IFN)-y, IL-2, IL-12, and IL-23 [17]. 

Two phase 3 randomized clinical trials (DERMIS 1 and DERMIS 2) were conducted to assess the efficacy of roflumilast cream 0.3% used once daily for eight weeks among patients with chronic plaque psoriasis. Investigator Global Assessment (IGA) was used to evaluate the efficacy, with scores ranging between 0-4. Statistically significant IGA success was observed in a greater percentage of patients treated with roflumilast compared to those treated with vehicle. Roflumilast cream demonstrated good efficacy and was well tolerated [18].

Both tapinarof and roflumilast are novel non-steroidal topical treatment options that are safe for regular long-term use on all affected areas, including sensitive or intertriginous areas, for maintaining remission. However, further research is necessary to evaluate the efficacy of these new topical therapies compared to other active psoriasis treatments, in order to assess long-term effects and safety profiles.

Topical Retinoids

Tazarotene, the most commonly prescribed topical retinoid, is used to treat psoriasis and can help maintain remission. It reduces keratinocyte hyperproliferation and associated redness. The most common side effect is skin irritation, which has limited its use in recent years. The efficacy of topical retinoids for the treatment of psoriasis is enhanced when combined with topical corticosteroids. Tazarotene increases epidermal thickness, thus reducing the risk of steroid-induced skin atrophy when combined with a topical corticosteroid.

Dithranol 

Dithranol is one of the oldest available treatments for psoriasis, and its mechanism of action has a direct anti-proliferative effect on epidermal keratinocytes. It is very effective when used in conjunction with phototherapy (ultraviolet B (UVB)) for inpatient or day treatment of psoriasis. Its use is limited by cosmetic unacceptability. Significant improvement, such as flattening of plaques, is usually noted around four weeks following initiation of treatment.

Coal Tar

Coal tar is an old but effective topical treatment for psoriasis, helpful in reducing symptoms of itching, inflammation, and scaling. Irritation of perilesional skin, folliculitis, and staining of clothes are common side effects of topical coal tar. Additionally, it can be considered malodorous, which often leads to poor patient compliance.

Difficult-to-treat areas

Scalp

Scalp psoriasis is often mistaken for seborrheic dermatitis, and while they can co-exist, psoriatic plaques are well-demarcated. Pruritus is very common in scalp psoriasis, and repetitive scratching may lead to the development of new lesions, a phenomenon known as Koebnerization. Management can be challenging due to the difficulty of directly applying products to the scalp. Patients may find applying creams or greasier products difficult to comply with; therefore, recognizing the patient's preferences is critical to encourage adherence and improve clinical outcomes.

Treatment modalities available for scalp psoriasis vary and often involve the use of potent or very potent topical corticosteroids, which have been found to be more effective than vitamin D analogs [18]. The combination of both corticosteroids and vitamin D analogs yields the best results. Therefore, combination therapy is preferred over monotherapy in the treatment of scalp psoriasis. Other options include coal tar preparations, tar-containing shampoos, and shampoos and emulsions containing salicylic acid, which are helpful adjuncts due to their keratolytic effects.

Flexures

Flexural psoriasis, sometimes called inverse psoriasis, affects body folds, which are considered humid areas. This reduces the formation of scales and can lead to misdiagnosis. The areas typically involved are the axillary, perineum, umbilicus, and inframammary regions. It can be particularly troublesome when the patient sweats. The mainstay of treatment is a potent topical corticosteroid. In resistant cases, a topical vitamin D analog or topical calcineurin inhibitor can be used. Topical roflumilast and tapinarof creams, as previously mentioned, are also used off-label for flexural psoriasis.

Nails

Treatment of nail psoriasis is challenging and often ineffective. First-line topical treatment options include very potent corticosteroids under occlusion or topical vitamin D3 analogs. Local injection of triamcinolone is effective but extremely painful and poorly tolerated. Therefore, to achieve a significant improvement, phototherapy or systemic treatment may be necessary. 

Phototherapy

**Figure 2:**
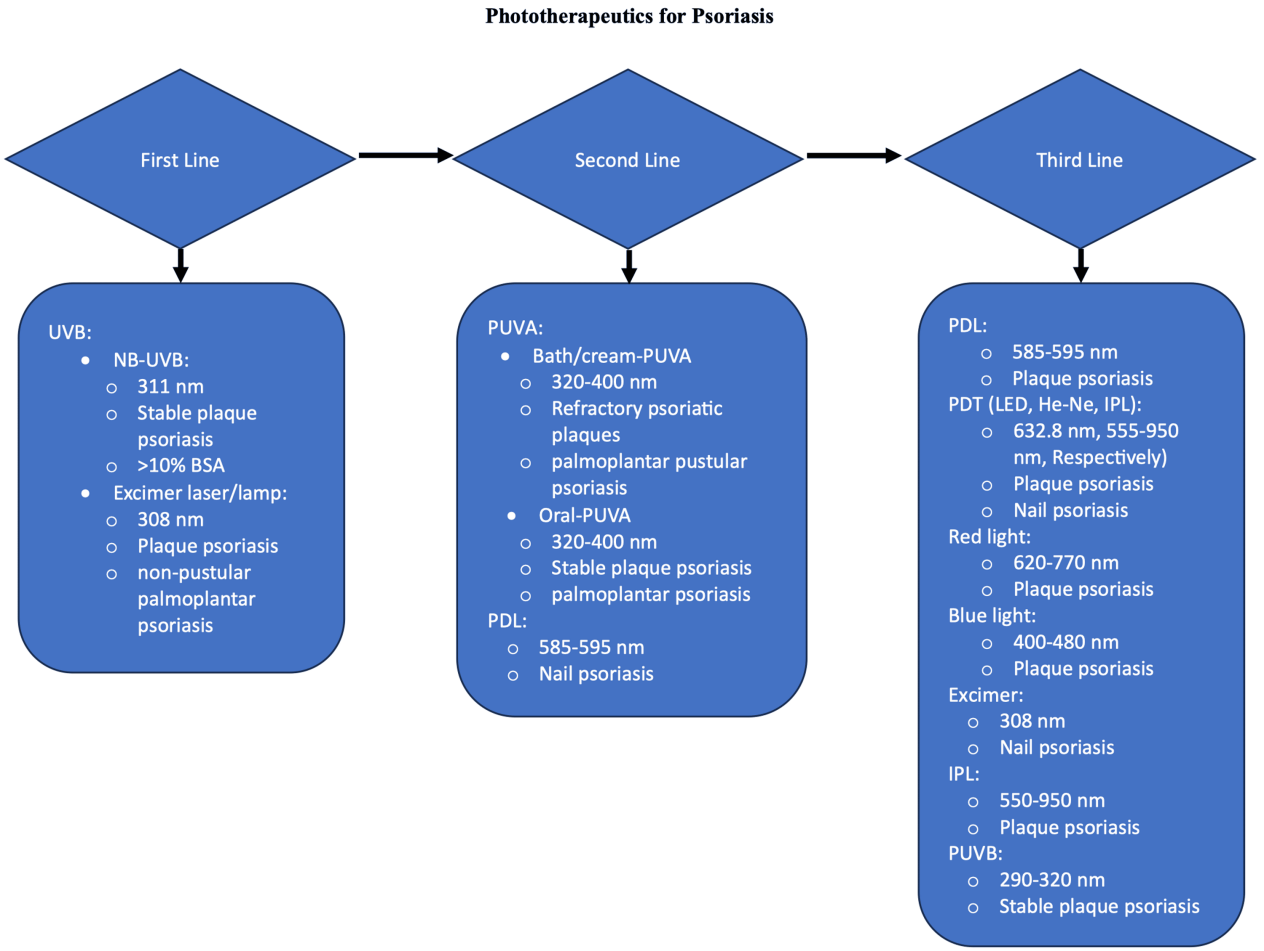
Phototherapy as first, second, and third line treatment for psoriasis Phototherapy options as a treatment for psoriasis. NB-UVB: Narrowband ultraviolet B; BSA: Body surface area; PUVA: Psoralen ultraviolet A; PDL: Pulsed dye laser; PDT: Photodynamic therapy; LED: Light-emitting diodes; He-Ne: Helium-Neon; IPL: Intense pulsed light; PUVB: Psoralen ultraviolet B Credits: Author Hasan Ashkanani

Phototherapy has been widely used to treat stable plaque psoriasis over the past several decades. It is an effective and safe treatment option without systemic side effects. A variety of light mechanisms of action have been developed to treat psoriasis, including UVB, psoralen ultraviolet A (PUVA), pulsed dye laser (PDL), photodynamic therapy (PDT), intense pulsed light (IPL), and others (Figure 2). Broadband ultraviolet B light (BB-UVB, 290-320 nm) was developed initially but was later replaced by narrowband ultraviolet B (NB-UVB, 311 nm) because it is more effective. Additionally, NB-UVB allows higher doses of UVB to be delivered with fewer side effects, such as burning.

Currently, the most commonly prescribed light therapy used to treat plaque psoriasis is NB-UVB therapy, which is primarily used for stable plaque psoriasis. NB-UVB has also been found to shorten the treatment time for pustular or erythrodermic psoriasis. For localized psoriasis, laser therapy, such as excimer laser therapy, is preferred over whole-body therapy because it spares uninvolved areas from UV radiation, allowing higher, targeted doses of UVB. This speeds up the response and minimizes any risks to the spared areas. An inappropriate choice of type or unnecessary exposure to phototherapy can cause side effects, such as erythema and skin burning. Physicians should exclude patients who are intolerant to light, have photosensitive reactions, are pregnant, or are taking certain drugs (e.g., sulfonamides and fluoroquinolones). Other short-term side effects of phototherapy include pruritus, pain, blisters, and crusting, which can occur anytime within the first 24 hours post-treatment. Phototherapy can be used as monotherapy or combined with biological agents for treating severe psoriasis [19]. PDL is used for psoriatic lesions in small areas, such as the nails, where it can provide optimal outcomes compared to other treatment options. Moreover, PDL has a specific wavelength that targets the chromophore hemoglobin and selectively damages vessels, making it the treatment of choice for congenital and acquired vascular lesions [20,21]. Although IPL is rarely used for the treatment of psoriasis, PDT-IPL has been found to have moderate curative effects on nail psoriasis.

Photochemotherapy (PUVA)

If NB-UVB fails to provide improvement, PUVA, either alone or combined with topical medications, can be used. Photochemotherapy (PUVA) is an effective treatment for psoriasis, consisting of a photosensitizing drug (psoralen) and long-wave ultraviolet radiation (UVA, 320-400 nm). It can be given systematically (oral/injections) or through bath/cream PUVA, both of which have been used to treat plaque psoriasis in the stationary phase or refractory psoriatic plaques. Clearance rates can reach 85-90%, with a mean remission length of six months. Side effects of PUVA include burning and pruritus. After PUVA therapy sessions, it is essential to wear UVA-protective glasses for at least 12 to 24 hours, as psoralen makes the eyes highly sensitive to UVA light. Increased sensitivity to UVA can cause cataract formation over time and lead to ocular damage. In palmoplantar pustular psoriasis, PUVA shows higher curative effects than UVB. Both NB-UVB and PUVA have the advantages of large radiation size and high efficacy. PUVA therapy is effective for chronic plaque psoriasis and guttate psoriasis, and when combined with retinoids, it is useful in treating more severe forms of the condition, such as erythrodermic psoriasis and generalized pustular psoriasis.

Systemic therapy 

Systemic therapy is reserved for moderate-to-severe cases of psoriasis and for patients who have not responded to topical treatments and phototherapy. Systemic options vary widely and include broadly acting oral immunomodulators as well as newer biological treatments.

Methotrexate

Methotrexate has been successfully used to treat psoriasis for over a decade. It is typically administered as a once-a-week, low-dose regimen. The usual dosage ranges from 7.5 to 25 mg per week when given by oral, intramuscular, intravenous (IV), or subcutaneous route. Serious side effects include bone marrow suppression and hepatic fibrosis, so monitoring of full blood counts is necessary. In patients with heavy alcohol use, the risk of hepatic fibrosis is increased.

When using methotrexate, concomitant 1 mg of folic acid supplementation is recommended to help reduce adverse effects, such as stomatitis and potential bone marrow suppression. Concomitant use of NSAIDs or co-trimoxazole with methotrexate may increase the risk of methotrexate toxicity. Methotrexate is teratogenic and mutagenic to spermatozoa; therefore, men and women planning to conceive should stop taking it at least three months prior to conception.

Methotrexate can be used for long-term treatment, in contrast to cyclosporine, which is associated with cumulative renal toxicity and is limited to short-term use.

Cyclosporine (T-cell Suppressor)

Cyclosporine is a calcineurin inhibitor that was initially used as an immunosuppressant medication in transplantation candidates to prevent organ rejection. Later, it was found to be effective in treating psoriasis by suppressing the effects of T-lymphocytes, thereby reducing IL-2 production. The typical dose range for cyclosporine therapy is 3-5 mg/kg daily, taken orally. Improvement is usually noted within four weeks.

Guidelines suggest that prior to starting therapy with cyclosporine, the patient should have normal blood pressure as well as normal renal and hepatic function. Uncontrolled hypertension, renal dysfunction, malignancies, known allergy or hypersensitivity to cyclosporine or its components, pregnancy, breastfeeding, and uncontrolled hyperkalemia and hyperuricemia, are considered absolute contraindications to cyclosporine therapy [22]. 

Due to an increased risk of developing skin malignancies, cyclosporine should be avoided in patients receiving phototherapy or photochemotherapy. The most common adverse effects associated with cyclosporine use include paresthesia, gingival hypertrophy, and hypertrichosis. Long-term use increases the risk of hypertension and nephrotoxicity, so treatment duration should not exceed six months at a time.

Retinoids

These medications are particularly beneficial for patients with severe forms of psoriasis, such as pustular, erythrodermic, or psoriasis related to HIV. Acitretin is a third-generation, polyaromatic retinoid that has an anti-proliferative effect and works primarily on the keratinocytes. It is taken orally, typically at a dose of 25 mg dose or 50 mg every other day. The full benefit of acitretin is usually noticed three to six months after starting the medication, due to its slow onset of effect [22].

Acitretin has a relatively safe treatment profile in long-term use, as it is not immunosuppressive. Guidelines suggest obtaining baseline blood tests, including liver function tests, lipid profiles, complete blood count, renal function tests, and pregnancy tests for female patients of childbearing age. These tests should also be done for routine monitoring during treatment.

Teratogenicity is the main side effect of acitretin; therefore, it should not be given to women of childbearing age or those who wish to conceive [22]. Female patients taking acitretin must avoid pregnancy for at least three years after discontinuation, whereas for isotretinoin, pregnancy must be avoided for one month after stopping the medication.

Oral Small Molecule Apremilast

The advanced small molecule apremilast is approved for the treatment of chronic plaque psoriasis in adults. It acts by inhibiting PDE-4, which subsequently increases the level of intracellular cyclic adenosine monophosphate (cAMP), downregulating the inflammatory responses of Th1, Th17, and type 1 IFN pathways [23]. Also, its ability to regulate levels of anti-inflammatory cytokines, such as IL-10, further helps improve psoriasis outcomes.

This treatment is reserved for patients who have not responded to phototherapy or systemic therapies such as cyclosporine and methotrexate. The recommended dosage for apremilast begins with 10 mg on day one, followed by a seven-day dose titration until the therapeutic dose of 30 mg twice daily is reached. The most commonly reported side effects include diarrhea, nausea, weight loss, and upper respiratory tract infections. Apremilast should be used with caution in patients with a history of depression. It is also approved and effective for the treatment of psoriatic arthritis.

Two 16-week, randomized trials supported the initial approval of apremilast for the treatment of moderate-to-severe psoriasis. In these trials, 30 mg of apremilast twice daily or a placebo was administered to 1,257 individuals with moderate-to-severe psoriasis. In the first trial, 33% of 562 patients treated with apremilast achieved PASI 75, compared to just 5% of 282 patients in the placebo group. Results from the other randomized trial were similar; data showed good results with apremilast as a treatment for patients with mild-to-moderate psoriasis. This study involved 595 patients with mild-to-moderate psoriasis who were randomly assigned to receive either apremilast 30 mg twice daily or a placebo. At week 16, 22% of patients in the apremilast group achieved a PGA score of 0 or 1 (clear or almost clear skin), with at least a two-point reduction from baseline, compared to only 4% of patients in the placebo group [23]. In addition, 33% of patients in the apremilast group achieved PASI 75 at week 16, compared to only 7% in the placebo group [23].

Deucravacitinib

In September 2022, the FDA-approved deucravacitinib, a first-in-class oral, selective, allosteric tyrosine kinase 2 (TYK2) inhibitor, after it demonstrated a favorable safety profile and greater efficacy than other available oral treatment options for plaque psoriasis. It is indicated for adults with moderate-to-severe plaque psoriasis who are candidates for systemic therapy or phototherapy.

A 52-week, double-blind, phase 3 clinical trial (POETYK PSO) compared the efficacy and safety of deucravacitinib, placebo, and apremilast [24]. Subjects were randomized to receive deucravacitinib 6 mg daily (n=511), placebo (n=255), or apremilast 30 mg twice a day (n=254). At week 16, significantly more patients in the deucravacitinib group (53%) achieved ≥75% reduction from baseline (PASI 75) compared to placebo (9.4%) and apremilast (39.8%) patients (P<0.0001 versus placebo and P=0.0004 versus apremilast). Additionally, 49.5% of patients in the deucravacitinib group achieved a PGA score of 0 or 1, compared to 8.6% in the placebo group and 33.9% in the apremilast group (P<0.0001 for both placebo and apremilast). In summary, deucravacitinib showed significantly higher response rates and better efficacy compared to placebo and apremilast. Moreover, it was well tolerated, with adverse event rates similar to those of placebo and apremilast. Serious adverse events were infrequent.

Biological treatment

Biologics are monoclonal immunoglobulin G (IgG) molecules (with the exception of etanercept) that target specific cytokines or receptors involved in the pathogenesis of psoriasis. They are administered through subcutaneous injections or IV infusion. Biologics are ushering in a new era of psoriasis treatment due to their excellent short-term and long-term efficacy and favorable tolerability. However, conventional systemic therapies are still widely used, either as monotherapy or in combination with biologics, due to their lower cost, availability, and easier route of administration. 

While biologics have revolutionized psoriasis treatment, there are long-term side effects associated with their use. Risk assessment should begin at the first visit by establishing comorbidities that may preclude the use of certain biological treatments. Infection is one of the main reasons for biologic discontinuation and due to the potential risk of tuberculosis (TB) acquisition or reactivation, clinical practice guidelines frequently recommend screening with an IFN-y release assay prior to starting biological therapy [25]. Physicians should therefore consider avoiding certain medications in patients at high risk for infection. Patients receiving biological therapy in combination with methotrexate are at an increased risk for herpes zoster [26]. Additionally, the coexistence of psychiatric comorbidity should not influence the choice of biological treatment, as it has been found that initiating a biological therapy may actually improve the patient’s psychiatric symptoms at first. While inflammatory bowel diseases (IBDs) can be treated with anti-TNF agents and ustekinumab, IL-17 antagonists may cause new-onset or paradoxically flare a pre-existing IBD. This was first noted in clinical trials of anti-IL-17 in patients with IBDs [27]. A balance of benefit versus risk should be applied in clinical practice.

Biologics approved by the FDA for the treatment of psoriasis include inhibitors to TNF-α, such as certolizumab, adalimumab, infliximab, and etanercept. Other biologics inhibit cytokines, such as the p40 subunit of the cytokines IL-12 and IL-23 (ustekinumab), IL-17 (brodalumab, ixekizumab, and secukinumab), and the p19 subunit of IL-23 (risankizumab, tildrakizumab, guselkumab) (Table 1). Biosimilars of TNF blockers are also FDA-approved for the treatment of plaque psoriasis. 

**Table 1:** Biological treatment approved by the FDA for psoriasis treatment FDA-approved biologics, classes, route, and date of approval are described in the table. TYK2: Tyrosine-kinase 2; TNF: Tumor necrosis factor; IL: Interleukin; AhR: Aryl-hydrocarbon receptor; PDE: Phosphodiesterase; FDA: Food and Drug Administration Credits: Author Sarah Qasem

Latest FDA-Approved Psoriasis Treatments			
Drug	Class	Route	Date of Approval
Deucravacitinib	TYK2 inhibitor	Oral	Sep/2022
Roflumilast	PDE-4 inhibitor	Topical	Jul/2022
Tapinarof	AhR modulating agent	Topical	May/2022
Risankizumab-rzaa	IL-23 antagonist	Subcutaneous	Apr/2019
Certolizumab pegol	TNF blocker	Subcutaneous	May/2018
Tildrakizumab-asmn	IL-23 antagonist	Subcutaneous	Mar/2018
Guselkumab	IL-23 antagonist	Subcutaneous	Jul/2017
Brodalumab	IL-17 antagonist	Subcutaneous	Feb/2017
Ixekizumab, secukinumab	IL-17 antagonist	Subcutaneous	Mar/2016, Jan/2015
Apremilast	PDE-4 inhibitor	Oral	Sep/2014
Ustekinumab	IL-12 and IL-23 antagonist	Subcutaneous	Sep/2009
Adalimumab	TNF blocker	Subcutaneous	Jan/2008
Infliximab	TNF blocker	IV infusion	Sep/2006
Etanercept	TNF blocker	Subcutaneous	Apr/2004

Biologics that inhibit TNF-α, p40 IL-12/23, and IL-17 are also approved for the treatment of psoriatic arthritis. An IL-36 receptor monoclonal antibody, spesolimab, is FDA-approved to treat generalized pustular psoriasis.

The FDA-approved biologics for pediatric patients with moderate-to-severe psoriasis include etanercept and adalimumab for children four years and older, ustekinumab, secukinumab, and ixekinumab for children six years and older, and certolizumab pegol for adolescents 18 years and older. Other promising treatments for pediatric psoriasis are undergoing phase 3 trials, including brodalumab, guselkumab, risankizumab, tildrakizumab, and deucravacitinib. 

TNF-α Inhibitors

Etanercept: The TNF-α inhibitor etanercept is FDA-approved for the treatment of adults and children aged four years and older with chronic moderate-to-severe plaque psoriasis, as well as for the treatment of psoriatic arthritis. The starting dose for adults is a 50 mg subcutaneous injection twice per week for three consecutive months, followed by a 50 mg injection once per week as maintenance therapy. In pediatric patients, the dose is 0.8 mg/kg as a weekly injection, with a maximum dose of 50 mg per week. 

In a 24-week, double-blinded, randomized etanercept trial, 652 adult individuals with active plaque psoriasis affecting at least 10% of BSA participated. A low dose of etanercept (25 mg once weekly), a medium dose (25 mg twice weekly), or a high dose (50 mg twice weekly) was administered randomly to the subjects. By week 12, 4% of patients in the placebo group, 14% of patients in the low-dose etanercept group, 34% of patients in the medium-dose etanercept group, and 49% of patients in the high-dose etanercept group had improved from baseline and reached PASI 75 (P<0.001 for all three comparisons with the placebo group). As treatment continued, the clinical results continued to improve. At week 24, 25% of patients in the low-dose group, 44% of patients in the medium-dose group, and 59% of patients in the high-dose group achieved at least PASI 75. In summary, subcutaneous etanercept (25 mg weekly, 25 mg twice weekly, and 50 mg twice weekly) was found significantly superior to placebo [28,29].

Infliximab: The TNF-α inhibitor infliximab is used in adults and children over the age of six years with moderate-to-severe plaque psoriasis. It has a rapid onset of action compared to other available biological agents. The standard dosing is 5 mg/kg for IV infusion, given over 2-3 hours at weeks 0, 2, and 6. Maintenance infusions are given at eight-week intervals.

A double-blinded, multicenter, phase 3 trial enrolled and randomized 378 patients with moderate-to-severe psoriasis to receive either an IV infusion of 5 mg/kg infliximab or a placebo at weeks 0, 2, and 6, followed by every eight weeks until week 46. At week 10, 80% of patients receiving infliximab demonstrated a minimum of PASI 75 improvement from baseline and 57% achieved at least PASI 90, compared to 3% and 1% improvement in the placebo group, respectively (P<0.0001). Statistically significant improvement in the infliximab group was noted [30]. Loss of efficacy over time occurred in 19% of patients due to the development of antibodies to infliximab. Infliximab is also effective for the treatment of psoriatic arthritis. 

Adalimumab: Adalimumab is a fully human monoclonal antibody with a high binding specificity against TNF-α; it was first used for rheumatoid arthritis and psoriatic arthritis. It is FDA-approved for the treatment of adults and children aged four years and older with moderate-to-severe chronic plaque psoriasis. The standard dose of adalimumab is a loading dose of 80 mg via subcutaneous injection at week 0, followed by 40 mg every other week starting at week 1.

The efficacy and safety of adalimumab for the treatment of moderate-to-severe chronic plaque psoriasis were further supported by REACH, a 16-week, randomized, double-blind trial. A total of 72 patients were randomized 2:1 to receive adalimumab (80 mg at week 0, followed by 40 mg every other week starting at week 1) or a matching placebo. At week 16, 31% of patients in the adalimumab group achieved a PGA score of clear or almost clear, compared to only 4% in the placebo group [30,31], suggesting that adalimumab is an effective and safe treatment.

Another randomized trial involving 147 patients with moderate-to-severe plaque psoriasis evaluated the results of adalimumab administered via subcutaneous injection at doses of 40 mg every other week, 40 mg weekly, and a placebo [31]. After 12 weeks, patients receiving adalimumab biweekly or weekly achieved PASI 75 at rates of 53% and 80%, respectively, compared to 4% in the placebo group. Improvements were sustained for 60 weeks.

Adalimumab may be used in patients who have failed to respond to etanercept or other biological therapies. Methotrexate can be added if there is psoriatic arthritis or to increase the efficacy of adalimumab.

Certolizumab pegol: Certolizumab pegol is a pegylated, recombinant, humanized antibody Fab fragment that blocks TNF-α. It is FDA-approved for the treatment of adults with moderate-to-severe psoriasis who are candidates for systemic therapy or phototherapy. Additionally, it is also effective for the treatment of psoriatic arthritis. The standard dosing is a 400 mg subcutaneous injection given every other week. It is considered the treatment of choice in pregnancy and should be considered first-line in pregnant women and those planning conception. Unlike other anti-TNF therapies, certolizumab pegol does not pass the placenta or bind to the neonatal Fc receptor because it lacks the IgG Fc region. 

Side effects of TNF-α inhibitors: TNF-α inhibitors are generally well tolerated, but side effects are possible and may include infusion and injection site reactions, minor infections such as upper respiratory tract infections, lupus-like syndrome, hepatitis B virus (HBV) reactivation, liver function test abnormalities, or hepatotoxicity, particularly with infliximab use. Additionally, reactivation of TB is a side effect of TNF-α inhibitors; TNF-α plays a major role in host immune defense against mycobacterial infections, particularly in granuloma formation, which contains the mycobacteria and prevents bacterial dissemination. Therefore, a high level of suspicion is necessary throughout treatment with TNF-α inhibitors and for six months after drug discontinuation. Patients taking TNF-α inhibitors should not receive live or live-attenuated vaccinations four weeks prior to, during, and six months after discontinuation of biological therapy.

Other side effects include serious infections, including viral, bacterial, and fungal infections, as TNF-α plays a key role in macrophage and phagosome activation, as well as neutrophil recruitment [31]. 

There is a potential risk of development or worsening of pre-existing cardiac failure. TNF inhibitors are not recommended in patients with severe cardiac failure (New York Heart Association (NYHA) class III and IV). Malignancies associated with TNF-α inhibitors use include non-melanoma skin cancer, although there is no evidence of increased risk of malignancy with TNF antagonists. There may also be an association between TNF inhibitors and demyelinating diseases, so they should be avoided in patients with a history of or a first-degree relative with demyelinating disease. Although TNF inhibitors are commonly used to treat psoriasis, paradoxical skin reactions can rarely occur and may even trigger new-onset psoriasis.

Infliximab-induced infusion reactions may occur during the time of infusion. Symptoms include shortness of breath, urticaria, hypotension, flushing, and headache.

Inhibitors of IL-17 Pathway

Secukinumab: Secukinumab, an anti-IL-17A human monoclonal antibody, is FDA-approved for treating moderate-to-severe plaque psoriasis. It is administered as a 300 mg subcutaneous injection once weekly at weeks 0, 1, 2, 3, and 4, followed by 300 mg every four weeks. It is also FDA-approved for the treatment of psoriatic arthritis. 

Secukinumab's efficacy in the treatment of moderate-to-severe psoriasis was supported by two 52-week, phase III, placebo-controlled trials (ERASURE and FIXTURE). In the ERASURE trial, 738 participants were enrolled. At week 12, 82% of patients in the 300 mg secukinumab group achieved PASI 75 compared to 72% in the 150 mg secukinumab group and only 5% in the placebo group. The FIXTURE trial, which included a total of 1,306 participants, used similar doses of secukinumab. The conclusion of the trial was that secukinumab was superior to both etanercept and placebo. After 12 weeks, PASI 75 was achieved by 77% of patients in the 300 mg secukinumab group, 67% of patients in the 150 mg secukinumab group, 44% of patients in the etanercept group, and only 5% of patients in the placebo group [27,32].

Ixekizumab: Ixekizumab is a humanized monoclonal antibody that binds to and acts against IL-17A. It is administered via subcutaneous injection as a 160 mg loading dose, followed by an 80 mg dose every two weeks for 12 weeks. After 12 weeks, injections are given once monthly. 

In three phase 3 trials of ixekizumab (UNCOVER 1, UNCOVER 2, and UNCOVER 3), a total of 3,736 patients with moderate-to-severe psoriasis were assigned to receive either subcutaneous injections of placebo, 80 mg of ixekizumab biweekly after a loading dose, or 80 mg of ixekizumab every four weeks after a loading dose. PASI 75 was achieved by 89% of patients in the biweekly dosing group, 82% of patients in the four-week dosing group, and only 3.9% of patients in the placebo group. Results also showed that ixekizumab was superior to placebo and effective throughout 60 weeks of treatment (P<0.001 for all comparisons of ixekizumab with placebo) [33].

Brodalumab: The recombinant human immunoglobulin IgG2 monoclonal antibody brodalumab binds to and inhibits IL-17 receptor (IL-17RA). The standard dosing is a 210 mg subcutaneous injection given at weeks 0, 1, and 2, followed by the same dose every two weeks. The efficacy of brodalumab was further supported by two phase 3 trials (AMAGINE 2 and AMAGINE 3), in which patients with moderate-to-severe psoriasis were randomly assigned to receive brodalumab (210 mg or 140 mg every two weeks), ustekinumab (45 mg or 90 mg depending on body weight), or placebo. After 12 weeks, PASI 75 was achieved in 86% and 67% of patients in the brodalumab 210 mg and 140 mg doses, respectively, compared to 6% of patients in the placebo group [34]. Brodalumab is contraindicated in patients with active Crohn’s disease [34].

Bimekizumab: The monoclonal IgG antibody bimekizumab works by neutralizing IL-17A, IL-17F, and IL-17A/F. It is FDA-approved for the treatment of moderate-to-severe psoriasis in adults who are candidates for systemic therapy. The safety and efficacy of bimekizumab have been evaluated by multiple clinical trials, comparing it to placebo, ustekinumab, adalimumab, and secukinumab.

One example is the BE SURE trial, a phase 3, multicenter, double-blind trial, which compared bimekizumab to adalimumab over a 56-week period [35]. A total of 478 patients were randomized to receive bimekizumab 320 mg once every four weeks (n=158), bimekizumab 320 mg every four weeks up to week 16 and every eight weeks thereafter (n=161), or adalimumab 40 mg every two weeks up to week 24 and bimekizumab 320 mg every four weeks thereafter (n=159). At week 4, a statistically significant improvement in psoriasis was observed in the bimekizumab group compared to the adalimumab group, with 244 (76.5%) and 50 (31.4%) patients reaching PASI 75, respectively (P<0.001). At week 16, 275 (86.2%) subjects treated with bimekizumab achieved a PASI 90 response compared to 75 (47.2%) patients of the adalimumab group (P<0.001). Finally, a PASI 90 response was achieved by 134 (84.8%) patients of the bimekizumab four-weekly group, 133 (82.6%) subjects who received bimekizumab four-weekly and then eight-weekly, and 82 (81.8%) patients who switched from adalimumab to bimekizumab at week 56. Oral candidiasis and diarrhea were reported more frequently in the bimekizumab group compared to the adalimumab group [36].

Another clinical trial, BE RADIANT, compared the efficacy and safety of bimekizumab versus secukinumab in a 48-week phase 3 trial. A total of 743 patients were enrolled and randomized to receive either bimekizumab 320 mg once every four weeks (n=373) or secukinumab 300 mg weekly up to week 4 and then every four weeks thereafter (n=370). In addition, subjects in the bimekizumab group were further divided from week 16 into two groups to receive maintenance dosing either once every four weeks or once every eight weeks (n=147 and n=215), respectively. At week 16, PASI 90 was achieved by 319 (85.5%) patients treated with bimekizumab compared to 275 (74.3%) patients of the secukinumab group. At week 48, 83.6% and 70.5% of patients of the bimekizumab and secukinumab groups, respectively, achieved PASI 90. The safety was similar for both groups [37]. 

Side effects of IL-17 inhibitors: The most common side effects of IL-17 inhibitors include upper respiratory tract infections, nasopharyngitis, and mucocutaneous candidiasis. IL-17 inhibitors should be used carefully in patients with a history of IBD. New-onset Crohn’s disease and ulcerative colitis have been reported in patients using IL-17 inhibitors.

Inhibitors of IL-23 and Related Cytokines

Ustekinumab: Ustekinumab is a fully human monoclonal antibody with a high affinity for the p40 subunit of IL-12 and IL-23. It is administered subcutaneously as per a weight-dependent dosing schedule. If the weight is less than 100 kg, a 45 mg subcutaneous injection is given at weeks 0, 4, and every 12 weeks thereafter. On the other hand, if the weight is >100 kg, the dose is 90 mg at weeks 0, 4, and every 12 weeks thereafter. Reported side effects include upper respiratory tract infections, joint pain, cough, and headache. 

Guselkumab: Guselkumab is a fully human monoclonal antibody that binds to IL-23 and blocks its activity via its receptors. By blocking IL-23, guselkumab inhibits the release of pro-inflammatory cytokines such as IL-17A, IL-17F, and IL-22, thereby inhibiting their signaling. 

The p40 subunit is shared with IL-12, while the p19 subunit is specific to IL-23. The standard dose includes initial loading doses of 100 mg subcutaneous injections given at week 0 and week 4. After the loading doses, injections are administered once every eight weeks.

Tildrakizumab: Tildrakizumab is a human monoclonal IgG1 lambda antibody that has a high affinity and selectively binds the p19 subunit of IL-23 in dendritic cells and keratinocytes, thereby inhibiting its interaction with IL-23 receptors. In 2018, tildrakizumab was approved by the FDA for the treatment of moderate-to-severe plaque psoriasis in adult patients who are candidates for systemic therapy or phototherapy. Tildrakizumab is given as a 100 mg subcutaneous injection at weeks 0, 4, and every 12 weeks thereafter. The approval of tildrakizumab was further supported by data from the reSURFACE clinical trials [38].

The reSURFACE 1 and reSURFACE 2 trials are two randomized, double-blind, controlled, phase 3 trials conducted to investigate whether tildrakizumab is superior to placebo and etanercept for the treatment of chronic plaque psoriasis in adults aged 18 years and older. In reSURFACE 1 (n=722), patients were randomly assigned to receive 200 mg tildrakizumab (n=308), 100 mg tildrakizumab (n=309), or placebo (n=155). After 12 weeks, PASI 75 was achieved by 62% of patients in the 200 mg tildrakizumab group, 64% of patients in the 100 mg tildrakizumab group, compared to only 6% in the placebo group (P<0.0001 for comparisons of both tildrakizumab groups versus placebo). In reSURFACE 2 (n=1090), patients were assigned to receive 200 mg tildrakizumab (n=314), 100 mg tildrakizumab (n=307), placebo (n=156), and etanercept (n=313). At week 12, PASI 75 was achieved by 66% of patients in the 200 mg tildrakizumab group and 61% of patients in the 100 mg tildrakizumab group, compared to 6% of patients in the placebo group and 48% of patients in the etanercept group (P<0.0001 for comparisons of both tildrakizumab doses versus placebo; P<0.0001 for 200 mg tildrakizumab versus etanercept; P=0.0010 for 100 mg tildrakizumab versus etanercept). In conclusion, for the treatment of moderate-to-severe chronic plaque psoriasis, tildrakizumab 200 mg and 100 mg were found to be superior and efficacious compared to placebo and etanercept and were well tolerated. 

Risankizumab: Risankizumab-rzaa is a human monoclonal IgG1 antibody that selectively binds the p19 subunit of IL-23, inhibiting the release of pro-inflammatory cytokines and chemokines implicated in the pathogenesis of psoriasis. In 2019, it was approved by the FDA to treat moderate-to-severe plaque psoriasis in adults who are candidates for systemic therapy or phototherapy, as well as to treat active psoriatic arthritis in adults. The recommended dosage is a 150 mg subcutaneous injection administered at weeks 0, 4, and every 12 weeks. Approval of risankizumab-rzaa was initially based on results from AbbVie’s global phase 3 clinical program, which included four randomized, double-blind clinical trials called UltIMMa 1, UltIMMa 2, IMMhance, and IMMvent. A total of 2,109 patients with moderate-to-severe plaque psoriasis were enrolled; the co-primary endpoints were a 90% reduction in PASI 90 and a static PGA (sPGA) score of either 0 (clear) or 1 (almost clear) at 16 weeks. In the IMMvent trial, risankizumab was found to be superior to and showed significantly greater efficacy than adalimumab. At week 16, approximately 72% of risankizumab-treated patients achieved PASI 90, compared to 47% of those treated with adalimumab (P<0.0001) [39]. 

An additional newly FDA-approved drug relevant to our topic is spesolimab, an IL-36 receptor inhibitor, which was found to be effective specifically in generalized pustular psoriasis. 

**Figure 3:**
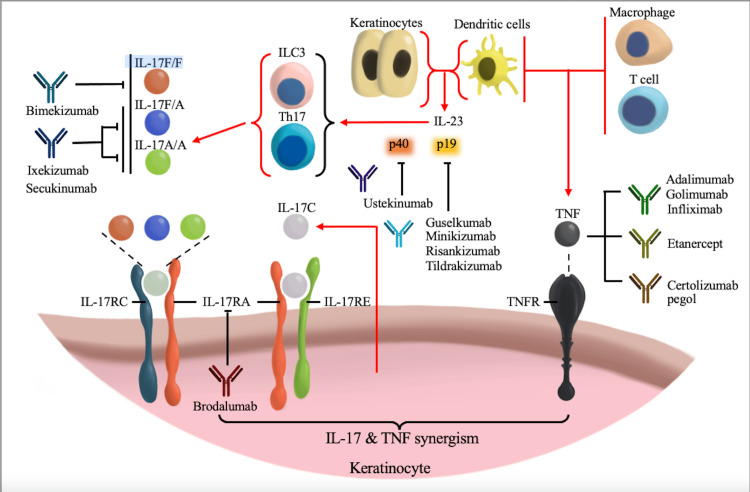
Biologics used in the management of psoriasis Biologics with their targets, including IL action sites and TNF. TNF: Tumor necrosis factor; TNFR: Tumor necrosis factor receptor; IL: Interleukin; Th17: T-helper 17 cell; ILC3: Innate lymphoid cell type 3 Credits: Author Ali Ali

A summative figure describing all the previously mentioned biological medications and their target sites in the treatment of psoriasis is shown in Figure 3.

## Conclusions

The management of psoriasis has entered an exciting new phase, driven by breakthroughs in novel therapies that are transforming how this complex condition is treated. Innovative options, such as biologics, selective tyrosine kinase inhibitors, and non-steroidal topicals like tapinarof and roflumilast, are providing new hope for patients, particularly those with moderate-to-severe disease. These treatments are not only more effective but also safer and better targeted, offering the potential to significantly improve the quality of life for individuals living with psoriasis.

By addressing specific pathways in the disease’s immune response, these therapies demonstrate the power of precision medicine. They highlight the value of tailoring treatments to meet individual patient needs, considering both the physical and psychological burden of psoriasis. As we move forward, it is critical to build on this progress through further research into long-term outcomes, combination approaches, and broader accessibility. The recent wave of FDA-approved treatments underscores a clear message: the future of psoriasis management lies in innovation, offering patients not just control over their symptoms but the possibility of thriving beyond the disease.
